# Effects of prediction and attention on tactile precision in somatosensory gating

**DOI:** 10.3758/s13414-026-03288-7

**Published:** 2026-06-11

**Authors:** Pierangelo N. D’Onofrio Pacheco, Eckart Zimmermann

**Affiliations:** Institute of Experimental Psychology, Heinrich-Heine University Düsseldorf, Building 23.02, Level 01, Room 41, Universitätsstrasse 1, 40225 Düsseldorf, Germany

**Keywords:** Goal-directed movements, Attention: space-based, Perception and action

## Abstract

Tactile sensitivity is reduced when the limb is in motion, a phenomenon known as somatosensory gating. In a previous study, we demonstrated that discrimination precision but not perceived intensity differed between active and passive movements. Here, we asked whether and how spatial attention modulates tactile precision in active and passive movements. Participants judged the relative intensity of two vibrations while the arm was still, actively moved, or passively transported by a movable platform. Visual attention was directed either to the movement start or goal position. Perceptual bias was reduced during both active and passive movement, independent of attentional allocation. In contrast, precision remained stable during active movement but declined during passive movement when attention was directed to the movement start. However, when attention was focused on the movement goals, precision was also high when doing passive movements. These findings indicate that during active movements, predictions based, likely on an efference copy, ensure tactile precision, whereas passive movements require spatial attention directed to the movement goal.

## Introduction

Tactile sensitivity declines when the limb is in motion, a phenomenon commonly referred to as somatosensory gating. This reduction in perceived intensity has been attributed to both central mechanisms, such as motor preparation and command signals, and peripheral factors, such as reafferent masking (Angel & Malenka, 1982; Chapman et al., 1987; Williams & Chapman, 2002). Early electrophysiological studies reported reduced amplitudes of somatosensory-evoked potentials (SEPs) around movement onset, suggesting that gating operates at both spinal and cortical levels (Seki & Fetz, 2012; Starr & Cohen, 1985).

In our previous work (D’Onofrio Pacheco & Zimmermann, 2026), we demonstrated that reductions in perceived intensity during active movement are statistically indistinguishable from those observed during passive movement. Crucially, however, tactile discrimination sensitivity differed between these conditions: Precision was preserved during active movements but reduced during passive movements. Additional experiments showed that the predictability of movement kinematics can account for this difference, because when participants could anticipate the velocity of passive motion, the brain could generate more accurate predictions about, forthcoming sensory input, allowing it to separate task-relevant tactile signals from movement-related noise. These findings indicate that tactile gating comprises at least two separable components: a bias component, which reduces the perceived magnitude of tactile input during limb motion and is captured by the point of subjective equality (PSE), and a precision component, which determines how finely two tactile intensities can be discriminated and is captured by the just-noticeable difference (JND). The reduction in perceived intensity likely reflects attenuation at peripheral and early central relays that is not eliminated by efference copy or learned expectations. Discrimination, however, depends on the reliability of the sensory estimate, which is degraded by unpredictable movement-related variability. When the brain can anticipate, forthcoming kinematics through efference copy during active movement or through learned regularities during predictable passive movement it can discount the predictable component of movement-related noise, yielding a more precise readout of the tactile signal. Predictions therefore sharpen the internal representation (improving JND) while leaving the movement-related attenuation of perceived magnitude (PSE) largely intact.

A growing body of research further suggests that gating is modulated by contextual and cognitive factors. Manipulations of task relevance, timing, and sensory predictability can reduce or even reverse suppression, enhancing discrimination at behaviorally relevant locations (Colino et al., 2014; Gertz et al., 2017; Kilteni & Ehrsson, 2022; Press et al., 2023; Voudouris et al., 2019). Moreover, studies conducted with stationary limbs show that directing attention to a specific body location enhances tactile processing and modulates somatosensory cortical activity (Gherri et al., 2023; Sambo & Forster, 2011; Schweisfurth et al., 2014). Visual spatial attention can also bias tactile processing at corresponding external locations(Driver & Spence, 1998; Eimer, 2001), and visuo–tactile interactions are strongly influenced by attentional allocation across modalities (Badde et al., 2020; Göschl et al., 2014). Together, these findings suggest that perceptual precision during movement may depend not only on motor-based predictions but also on whether visual attention is directed to a spatial location that provides reliable information about the upcoming movement, thereby enhancing the predictability of its sensory consequences.

In this study, we tested the influence of overt visual attention on intensity estimates of tactile stimuli presented during limb movement. Participants directed gaze either to the start or to the goal location of a horizontal arm movement while tactile stimuli were delivered during active or passive displacement of the arm. In the active condition, detailed information about movement kinematics was available in the internal motor commands. An efference copy of these commands may be used to continuously predict and evaluate reafferent tactile input. In the passive condition, however, no efference copy is available, and the brain must rely on learned externally derived signals to infer movement kinematics. Although both proprioceptive and visual cues can contribute to this inference, vision typically provides a more reliable estimate of the movement endpoint (Ernst & Banks, 2002; Körding & Wolpert, 2004).

We therefore reasoned that directing gaze to the goal location would provide the visual system with direct spatial information about the movement goal. This enhanced spatial information could improve the brain's predictions about the ongoing passive movement and its sensory consequences. Conversely, directing gaze to the movement start position would draw attentional resources away from the goal and reduce the reliability of such spatial predictions. We hypothesized that perceived intensity, quantified by the PSE, would be attenuated in all movement conditions, reflecting a general movement-related bias, whereas discrimination precision, quantified by the JND, would remain high and attention-independent during self-generated movement but decline during passive movement unless attention was focused at the goal location.

## Methods

### Participants

Twenty-one right-handed adults were recruited, and two were excluded from the final analysis due to unusable psychometric functions and one did not complete the study, resulting in a final sample of eighteen participants (12 women; mean age = 22.2 years, *SD* = 3.1). All reported normal or corrected-to-normal vision, were right-handed, and had no history of neurological or psychiatric disorders. The study conformed to the Declaration of Helsinki and was approved by the ethics committee of the Faculty of Mathematics and Natural Sciences, Heinrich Heine University Düsseldorf.

### Apparatus and materials

Participants sat with their head stabilized by a chin rest 67 cm from a 24-inch monitor (1,920 × 1,080 px, 144 Hz refresh rate). Eye position was tracked monocularly (right eye) with a Tobii 5 eye tracker (250 Hz), mounted below the screen.

The right arm rested on an ergonomic support. In the active condition, participants moved a Touch X haptic stylus (3D Systems) along a 20-cm horizontal path between two visual targets. In the passive condition, the same stylus was attached to a custom linear rail driven by a stepper motor (Arduino Leonardo controller) that translated the hand at a constant velocity of 200 mm/s.

Tactile stimulation was delivered by a 1-cm vibrotactile actuator fixed over the median-nerve region of the right forearm. Each vibration lasted 300 ms. On every trial, the probe vibration had a fixed duty cycle of 55%, while the comparison vibration was randomly selected from nine possible duty cycles (10, 20, 30, 40, 50, 60, 70, 80, or 90%). Vibrations were triggered by an Arduino Nano microcontroller synchronized with the timing routines. Participants wore Soundcore Life Q30 noise-canceling headphones to mask auditory cues. Responses were given on a three-key keypad (Pimoroni Keybow). All experimental measurements including eye tracking, stylus tracking, stimulus timing, and response collection were controlled by custom C# scripts in a 3D environment.

### Design and procedure

The experiment followed a 3 (movement: control, active, passive) × 2 (attention: start, end) factorial within-subjects design. The order of movement blocks was counterbalanced across participants, and attention conditions were randomized between each block.

**Fig. 1:**
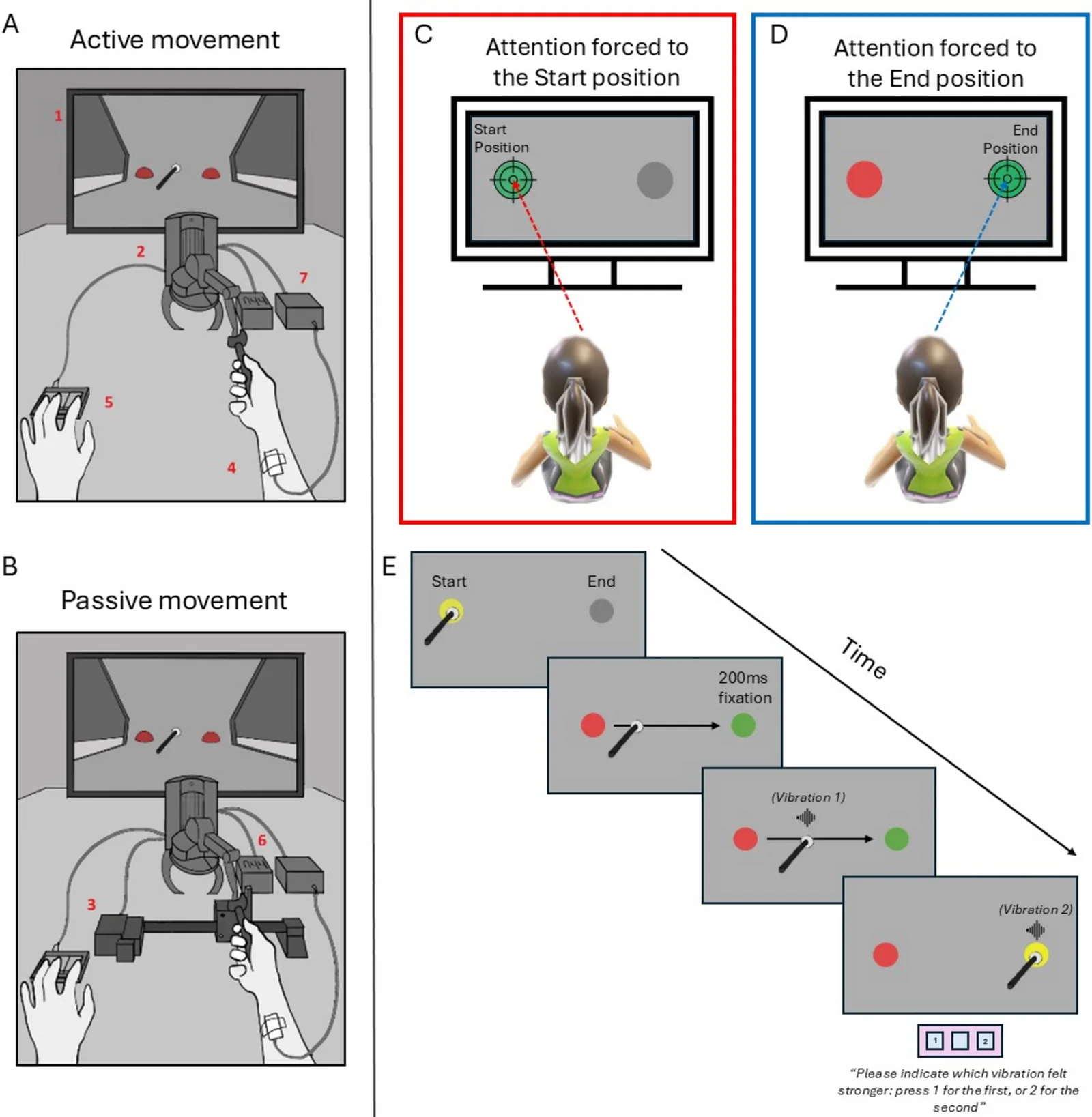
Experimental set-up and task design. **A** Schematic of the active task. Participants moved their right arm with a stylus from the first (left) to the second (right) sphere. A vibrotactile stimulus was delivered on the median nerve with a 200-ms delay, the same as for the passive condition). After reaching the second sphere, a second vibration was presented 200 ms later. Participants judged which vibration felt stronger by pressing a key (“1” = first stronger, “2” = second stronger) on the Pimoroni Keybow (5). **B** Active condition apparatus. The task display (1) guided the movement of a stylus tracked by the Touch X system (2). Vibrotactile stimulation was controlled by an Arduino Nano (7), with the actuator placed over the right median nerve (4). Responses were given via the Keybow. **C** Passive condition apparatus. The participant’s arm was moved by a rail system (3) driven by a stepper motor (Arduino Leonardo, 6). Passive movements (200 mm/s). Vibrotactile stimuli were delivered 200 ms after movement onset and again 200 ms after reaching the target sphere. (Color figure online)

In the control condition, participants' arms remained still throughout the entire condition. After the eye tracker confirmed that gaze was maintained within 1° of the cued fixation location for 200 ms, a probe vibration was delivered, followed 800 ms later by the comparison vibration. In the active movement condition, participants moved the stylus from the start position to the endpoint following the green cue. The probe vibration occurred 200 ms after movement onset, and the comparison vibration was delivered 200 ms after the stylus reached the endpoint (Fig. 1A). In the passive condition, the linear rail transported the participant’s arm along the same trajectory and velocity profile as in the active condition, and the timing of the vibrations was identical (Fig. 1B).

At the start of each trial, two colored spheres appeared on the screen corresponding to the start and endpoint of the horizontal movement path. One sphere changed color to indicate the location participants should fixate. The trial began once gaze was maintained within 1° of the cued location for 200 ms, at which point the fixation cue turned green to signal that the trial had started (see Fig. 1E). In the attention manipulation, the fixation cue appeared over the starting sphere (start condition, Fig. 1C) or the endpoint sphere (shifted condition, Fig. 1D).

On every trial, participants judged which of the two vibrations felt stronger by pressing "1" if the first vibration felt stronger or "2" if the second did. Each participant completed one block for each movement type, with 100 trials per block, resulting in a total of 500 trials including practice. The eye tracker was recalibrated between blocks whenever drift exceeded 0.5°.

### Data analysis

Responses were fitted with cumulative Gaussian psychometric functions using maximum-likelihood estimation for each participant and condition. The point of subjective equality (PSE) was used to indicate perceptual bias. The just-noticeable difference (JND) was given by the standard deviation of the fitted function, indexing discrimination precision. Trials with movement latency, duration, or path curvature exceeding ± 2 standard deviations from a participant's mean were excluded (< 5% of trials).

PSE and JND values were analyzed with separate repeated-measures analyses of variance (ANOVAs; movement × attention). Sphericity violations were corrected with Greenhouse–Geisser adjustments. Post hoc tests used Holm correction. Effect sizes are reported as partial η^2^ for ANOVAs. Analyses were performed in JASP 0.18.1.0, with α = 0.05.

## Results

### Bias (PSE)

**Fig. 2:**
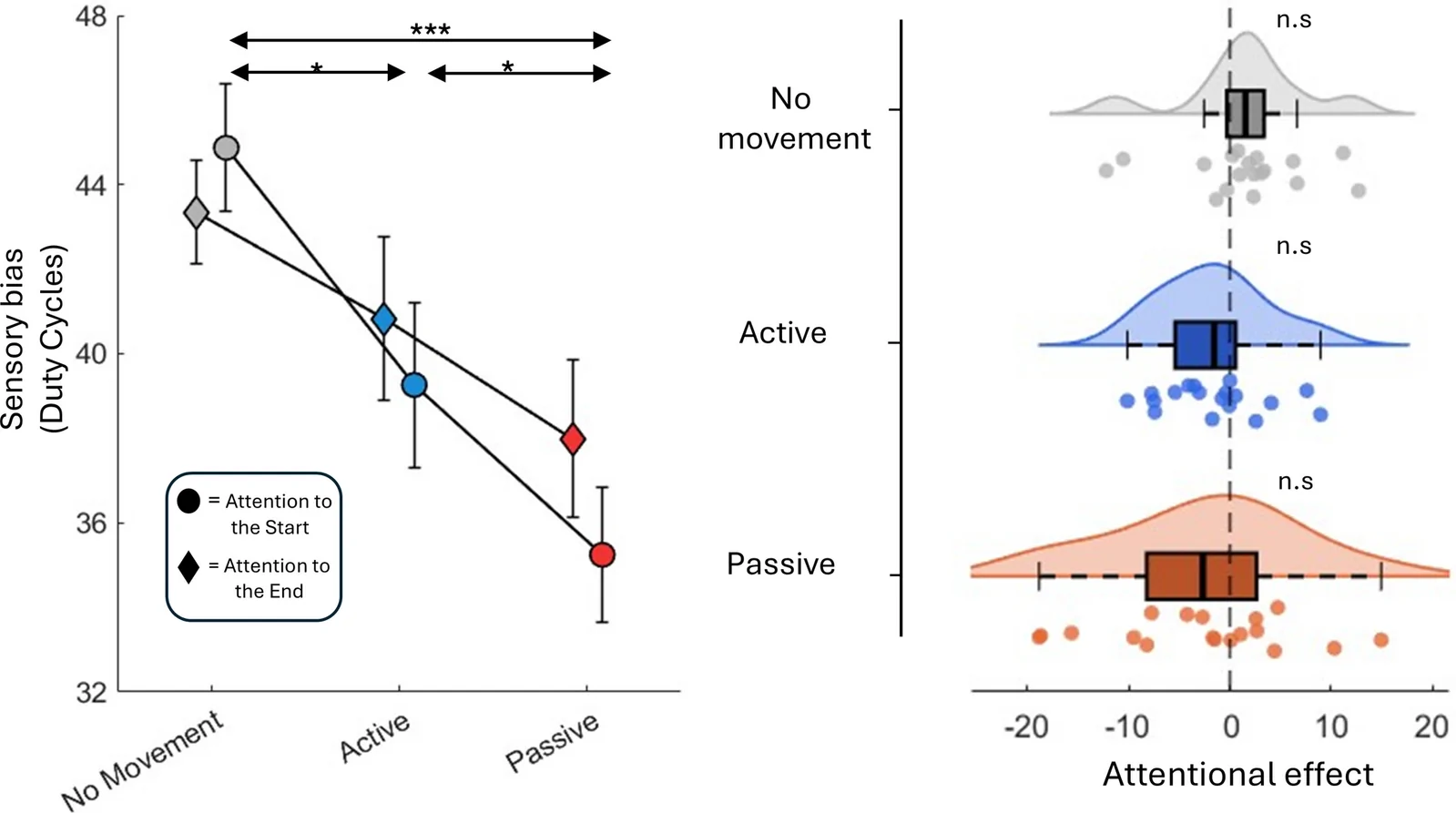
Effects of movement and attention on perceptual bias (PSE). Left: Mean PSE values (duty cycles) plotted as a function of the movement condition (no movement, active, passive) and attention. Circles indicate attention directed to the start position, and diamonds indicate attention directed to the end (goal) position. Error bars represent ± 1 *SEM*. Horizontal brackets denote significant post hoc comparisons from the 3 (movement) × 2 (attention) repeated-measures ANOVA. Right: Attentional effect on PSE expressed as the difference between End and Start fixation (Δ = end − start) for each movement condition. Half-violin plots show the distribution of individual values; boxplots represent median and interquartile range; dots indicate individual participants. Horizontal brackets denote significant post hoc comparisons for the main effect of movement condition, collapsed across the two attention conditions (start and end). Asterisks therefore refer jointly to both attention conditions, reflecting the absence of a significant Movement × Attention interaction on PSE. Asterisks denote significant post-hoc comparisons (*p <.05, ***p <.001) and “n.s.” denotes nonsignificant comparisons. (Color figure online)

A 3 (movement: control, active, passive) × 2 (attention: start, shifted) repeated-measures ANOVA (Fig. 2, left) revealed a main effect of movement, *F*(2,34) = 18.30, *p* <.001, ηₚ^2^ = 0.52, indicating reduced perceived intensity during movement. Neither the main effect of attention, *F*(1,17) = 0.77, *p* =.394, ηₚ^2^ = 0.04, nor the movement × attention interaction, *F*(2,34) = 1.59, *p* =.218, ηₚ^2^ = 0.09, reached significance.

Post hoc comparisons (Holm corrected) showed that both movement conditions yielded significantly lower PSEs than the stationary control condition. The active condition (*M* = 3.96, *SD* = 0.82) and the passive condition (*M* = 3.65, *SD* = 0.72) both produced smaller PSEs than control (*M* = 4.41, *SD* = 0.58), *t*(17) = 2.85, *p* =.022, and *t*(17) = 7.56, *p* <.001, respectively.

Although the marginal contrast suggested slightly lower PSEs in the passive than in the active movement, *t*(17) = 2.72, *p* =.022, this difference was not supported by the within-attention contrasts: Active-start versus passive-start and active-shift versus passive-shift were both nonsignificant (both *p* values =.477), whereas the cross-attention contrasts which confound movement type with attention allocation either failed to reach significance (passive-start vs. active-shift, *p* =.055) or showed no difference (active-start vs. passive-shift, *p* =.955). At matched attention, therefore, perceived intensity did not differ between active and passive movement.

There was no effect of overt attention. Collapsed across movement, mean PSEs were comparable for start fixation (*M* = 3.98, *SD* = 0.70) and shifted fixation (*M* = 3.90, *SD* = 0.71), *t*(17) = 0.88, *p* =.394.

### Precision (JND)

**Fig. 3:**
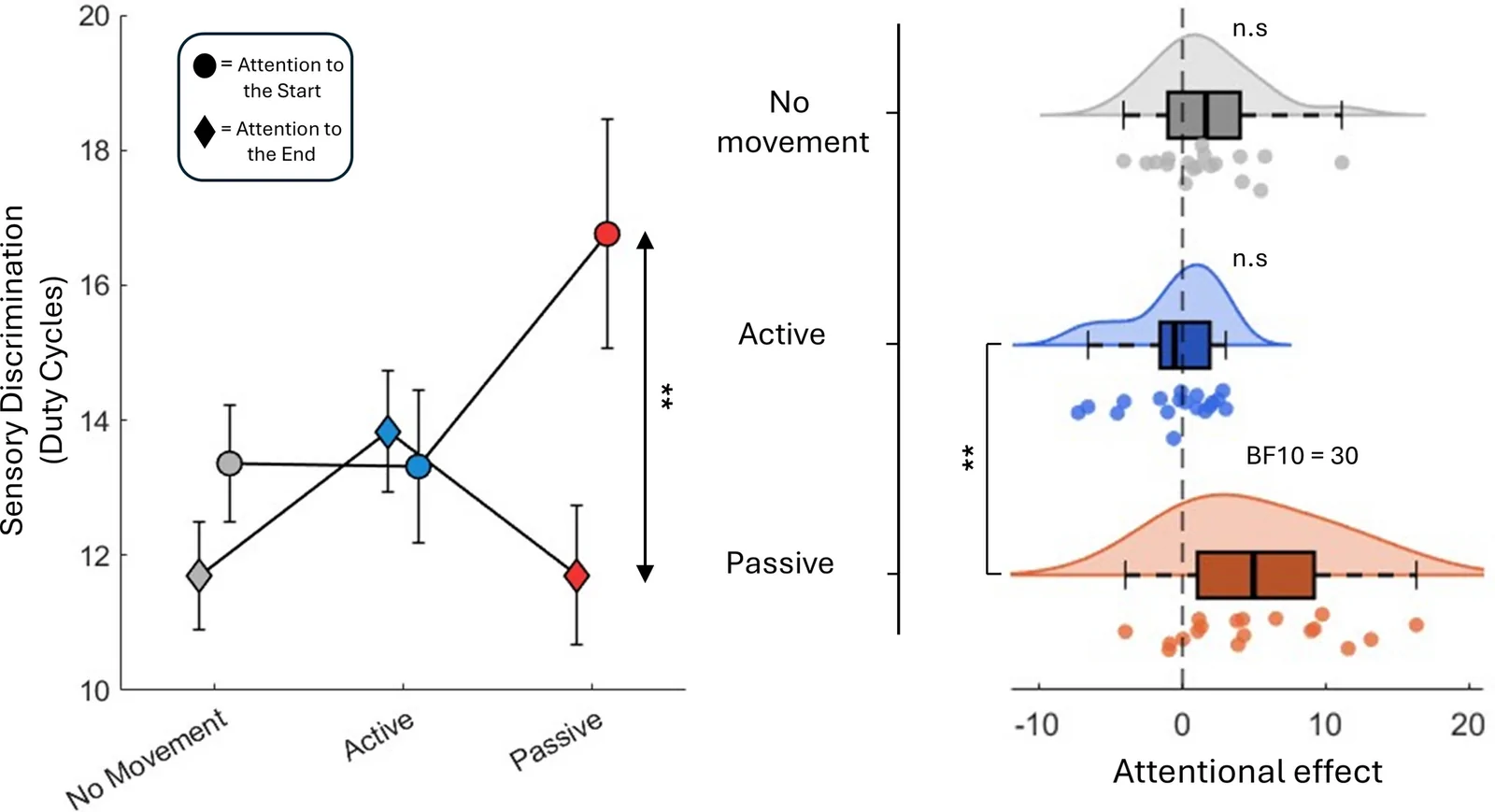
Effects of movement and attention on discrimination precision (JND). Left: Mean JND values (duty cycles) plotted as a function of movement condition (no movement, active, passive) and attention. Circles indicate attention directed to the start position, and diamonds indicate attention directed to the end (goal) position. Error bars represent ± 1 *SEM*. Vertical brackets denote significant comparisons from the 3 (movement) × 2 (attention) repeated-measures ANOVA. Right: Attentional effect on JND expressed as the difference between end and start fixation (Δ = end − start) for each movement condition. Violin plots depict the distribution of individual values; boxplots show median and interquartile range; dots represent individual participants. Bayesian factor (BF₁₀) is indicated where reported. (Color figure online)

The same 3 (movement: control, active, passive) × 2 (attention: start, shifted) ANOVA on JNDs (Fig. 3, left) revealed a main effect of attention, *F*(1,15) = 17.48, *p* <.001, ηₚ^2^ = 0.54, with higher precision when gaze was shifted to the movement endpoint than when it was centered at the start position. Importantly, a significant movement × attention interaction was observed, *F*(2,30) = 6.53, *p* =.004, ηₚ^2^ = 0.30, indicating that the attentional benefit differed across movement types.

Post hoc tests showed that during active movement, JNDs were low and unaffected by attention (start: *M* = 1.33, *SD* = 0.48; shifted: *M* = 1.38, *SD* = 0.38; *p* = 1.00). During passive movement, however, tactile precision depended strongly on gaze position (start: *M* = 1.67, *SD* = 0.72; shifted: *M* = 1.17, *SD* = 0.44; *p* =.019, BF₁₀ = 29.22). Notably, the passive-shifted condition reached the same precision level as the active conditions, indicating full recovery of discrimination when attention was directed to the goal.

In contrast, control trials at rest showed no significant attentional modulation (start: *M* = 1.34, *SD* = 0.37; shifted: *M* = 1.17, *SD* = 0.34; *p* =.692).

### Direct quantification of attentional modulation

A one-way repeated-measures ANOVA on the attentional difference scores (Δ = end − start) for JNDs confirmed a significant effect of movement condition, *F*(2,34) = 6.65, *p* =.004. Bonferroni-corrected post hoc comparisons revealed that the attentional benefit was significantly larger in the passive than in the active condition (*p* =.010; Fig. 3, right). A Bayesian paired* t* test further confirmed that attention improved precision within the passive condition (BF₁₀ = 29.11), providing strong evidence for an attentional effect on discrimination when movement was externally generated. No comparable attentional effect was found in the Active or Control conditions, and attentional manipulation did not influence PSE values in any condition (see Fig. 2, right).

## Discussion

The present findings address the mechanisms underlying somatosensory gating by demonstrating that movement differentially affects two aspects of tactile perception: perceptual bias and discrimination precision are differently modulated during limb motion. Consistent with our hypothesis, perceived intensity was attenuated during both active and passive movement, indicating that bias reflects a general movement-related modulation rather than a process specific to motor commands. In contrast, discrimination precision depended critically on the availability of predictive information. Precision remained stable during active movement regardless of attentional allocation, whereas during passive movement it declined unless attention was directed to the movement endpoint.

The different modulation of accuracy and precision directly supports the view that tactile gating is not a unitary suppression mechanism but the outcome of separable processes. The reduction in perceived intensity likely reflects both central gating signals linked to motor commands and peripheral factors such as sensory masking from movement-related afferent inflow (Angel & Malenka, 1982; Chapman et al., 1987; Williams & Chapman, 2002). Crucially, however, precision was preserved only when predictive information about movement kinematics was reliable. During active movement, internal motor commands generate an efference copy that supports forward prediction of reafferent tactile input (Wolpert & Flanagan, 2001), thereby stabilizing discrimination performance. During passive movement, in the absence of such motor-based prediction, precision became dependent on externally derived signals. This interpretation is consistent with recent demonstrations that central predictive mechanisms modulate tactile suppression beyond proprioceptive consequences (Altermatt et al., 2023; Arikan et al., 2024).

Our manipulation of gaze position demonstrates that directing visual attention to the spatial location toward which the limb is moving can compensate for the absence of an efference copy. Directing gaze to the movement endpoint likely increased the reliability of spatial predictions derived from visual input, restoring precision to the level observed during voluntary action. In contrast, maintaining attention at the movement start position reduced the availability of predictive information about the goal, leading to diminished discrimination sensitivity. These findings extend previous work showing that tactile processing is enhanced at attended locations and that visual attention can modulate tactile gain at spatially corresponding positions (Driver & Spence, 1998; Eimer, 2001; Sambo & Forster, 2011). Together with the preserved precision during active movement, these results point to two mechanisms for maintaining tactile fidelity during motion: an intrinsic, motor-based mechanism in which efference copy stabilizes sensory processing, and a extrinsic, cue-driven mechanism in which visual attention to the movement goal supplies the spatial predictions that are otherwise absent.

These findings have broader implications for understanding predictive control and agency. Within frameworks that link motor prediction to sensory processing (Friston, 2010; Press et al., 2023), our results suggest that the nervous system deploys at least two mechanisms to maintain tactile discrimination during movement: efference-based prediction during voluntary movement and attention-dependent compensation consistent with goal-directed attentional control (Corbetta & Shulman, 2002) when agency is absent. Conditions characterized by altered attentional allocation or impaired efference copy, as has been shown on the autistic continuum (Pomè & Zimmermann, 2024, 2025), may disrupt this balance. On the other hand, patients with functional neurological disorder, which experience movements as involuntary, show altered sensory attenuation alongside abnormal attention to the body (Brown et al., 2013). Our dual-mechanism framework predicts that when the intrinsic, motor-based mechanism is disrupted, tactile precision should become strongly dependent on where attention is directed during movement. In conclusion, the present results demonstrate that tactile gating is not a uniform suppressive process: motor-based prediction preserves discrimination through an intrinsic mechanism, whereas attention restores it through an extrinsic mechanism when prediction must be externally sustained.

## Data Availability

We provide trial-level data and mean result of singles participants. The datasets are available (10.5281/zenodo.18877381).

## Code availability

The code for the psychometric function fitting is available here: https://github.com/pierodonpac/Sensory-Gating-Analysis

## Declarations

### Open practices statement

The data and materials for this experiment are available online (10.5281/zenodo.18877381). The analysis code is available online (https://github.com/pierodonpac/Sensory-Gating-Analysis). The experiment was not preregistered.

## References

[CR1] Altermatt, M., Thomas, F. A., & Wenderoth, N. (2023). Movement predictability modulates sensorimotor processing. *Frontiers in Human Neuroscience,**17*, Article 1237407. 38053650 10.3389/fnhum.2023.1237407 PMC10694232

[CR2] Angel, R. W., & Malenka, R. C. (1982). Velocity-dependent suppression of cutaneous sensitivity during movement. *Experimental Neurology,**77*(2), 266–274. 7095061 10.1016/0014-4886(82)90244-8

[CR3] Arikan, B. E., Voudouris, D., Straube, B., & Fiehler, K. (2024). Distinct role of central predictive mechanisms in tactile suppression. *iScience,**27*, Article 110582. 10.1016/j.isci.2024.110582 39188983 10.1016/j.isci.2024.110582 PMC11345528

[CR4] Badde, S., Myers, C. F., Yuval-Greenberg, S., & Carrasco, M. (2020). Oculomotor freezing reflects tactile temporal expectation and aids tactile perception. *Nature Communications,**11*(1), Article 3341. 32620746 10.1038/s41467-020-17160-1 PMC7335189

[CR5] Brown, H., Adams, R. A., Parees, I., Edwards, M., & Friston, K. (2013). Active inference, sensory attenuation and illusions. *Cognitive Processing,**14*, 411–427. 10.1007/s10339-013-0571-3 23744445 10.1007/s10339-013-0571-3 PMC3824582

[CR6] Chapman, C. E., Bushnell, M., Miron, D., Duncan, G., & Lund, J. (1987). Sensory perception during movement in man. *Experimental Brain Research,**68*(3), 516–524. 3691723 10.1007/BF00249795

[CR7] Colino, F. L., Buckingham, G., Cheng, D. T., van Donkelaar, P., & Binsted, G. (2014). Tactile gating in a reaching and grasping task. *Physiological Reports,**2*, Article e00267. 10.1002/phy2.267 24760521 10.1002/phy2.267 PMC4002247

[CR8] Corbetta, M., & Shulman, G. L. (2002). Control of goal-directed and stimulus-driven attention in the brain. *Nature Reviews Neuroscience,**3*(3), 201–215. 11994752 10.1038/nrn755

[CR9] D’Onofrio Pacheco, P. N., & Zimmermann, E. (2026). Movement enhances tactile sensitivity through prediction. *Journal of Neurophysiology,**135*(4), 888–895. 10.1152/jn.00558.2025 41843461 10.1152/jn.00558.2025 PMC7619185

[CR10] Driver, J., & Spence, C. (1998). Cross–modal links in spatial attention. *Philosophical Transactions of the Royal Society of London. Series b: Biological Sciences,**353*(1373), 1319–1331. 9770225 10.1098/rstb.1998.0286 PMC1692335

[CR11] Eimer, M. (2001). Crossmodal links in spatial attention between vision, audition, and touch: Evidence from event-related brain potentials. *Neuropsychologia,**39*(12), 1292–1303. 11566312 10.1016/s0028-3932(01)00118-x

[CR12] Ernst, M. O., & Banks, M. S. (2002). Humans integrate visual and haptic information in a statistically optimal fashion. *Nature,**415*(6870), 429–433. 11807554 10.1038/415429a

[CR13] Friston, K. (2010). The free-energy principle: A unified brain theory? *Nature Reviews Neuroscience,**11*(2), 127–138. 20068583 10.1038/nrn2787

[CR14] Gertz, H., Voudouris, D., & Fiehler, K. (2017). Reach-relevant somatosensory signals modulate tactile suppression. *Journal of Neurophysiology,**117*, 2262–2268. 10.1152/jn.00052.2017 28250147 10.1152/jn.00052.2017 PMC5461667

[CR15] Gherri, E., White, F., & Venables, E. (2023). On the spread of spatial attention in touch: Evidence from event-related brain potentials. *Biological Psychology,**178*, Article 108544. 36931591 10.1016/j.biopsycho.2023.108544

[CR16] Göschl, F., Engel, A. K., & Friese, U. (2014). Attention modulates visual-tactile interaction in spatial pattern matching. *PLoS ONE,**9*(9), Article e106896. 25203102 10.1371/journal.pone.0106896 PMC4159283

[CR17] Kilteni, K., & Ehrsson, H. H. (2022). Predictive attenuation of touch and tactile gating are distinct perceptual phenomena. *iScience,**25*, Article 104077. 10.1016/j.isci.2022.104077 35372807 10.1016/j.isci.2022.104077 PMC8968059

[CR18] Körding, K. P., & Wolpert, D. M. (2004). Bayesian integration in sensorimotor learning. *Nature,**427*(6971), 244–247. 14724638 10.1038/nature02169

[CR19] Pomè, A., & Zimmermann, E. (2024). Visuo-motor updating in individuals with heightened autistic traits. *eLife,**13*, Article RP94946. 10.7554/eLife.94946 38913073 10.7554/eLife.94946 PMC11196106

[CR20] Pomè, A., & Zimmermann, E. (2025). Disrupted sensorimotor predictions in high autistic characteristics. *Proceedings of the National Academy of Sciences,**122*(17), Article e2501624122. 10.1073/pnas.2501624122 10.1073/pnas.2501624122 PMC12054823 40258147

[CR21] Press, C., Thomas, E. R., & Yon, D. (2023). Cancelling cancellation? Sensorimotor control, agency, and prediction. *Neuroscience & Biobehavioral Reviews,**145*, Article 105012. 10.1016/j.neubiorev.2022.105012 36565943 10.1016/j.neubiorev.2022.105012

[CR22] Sambo, C. F., & Forster, B. (2011). Sustained spatial attention in touch: Modality‐specific and multimodal mechanisms. *The Scientific World Journal,**11*(1), 199–213. 21258762 10.1100/tsw.2011.34 PMC5720045

[CR23] Schweisfurth, M. A., Schweizer, R., & Treue, S. (2014). Feature-based attentional modulation of orientation perception in somatosensation. *Frontiers in Human Neuroscience,**8*, Article 519. 25071535 10.3389/fnhum.2014.00519 PMC4095560

[CR24] Seki, K., & Fetz, E. E. (2012). Gating of sensory input at spinal and cortical levels during preparation and execution of voluntary movement. *Journal of Neuroscience,**32*(3), 890–902. 22262887 10.1523/JNEUROSCI.4958-11.2012 PMC3293372

[CR25] Starr, A., & Cohen, L. G. (1985). ‘Gating’of somatosensory evoked potentials begins before the onset of voluntary movement in man. *Brain Research,**348*(1), 183–186. 4063823 10.1016/0006-8993(85)90377-4

[CR26] Voudouris, D., Broda, M. D., & Fiehler, K. (2019). Anticipatory grasping control modulates somatosensory perception. *Journal of Vision,**19*, Article 4. 10.1167/19.5.4 31058990 10.1167/19.5.4

[CR27] Williams, S. R., & Chapman, C. E. (2002). Time course and magnitude of movement-related gating of tactile detection in humans. III. Effect of motor tasks. *Journal of Neurophysiology,**88*, 1968–1979. 10.1152/jn.2002.88.4.1968 12364522 10.1152/jn.2002.88.4.1968

[CR28] Wolpert, D. M., & Flanagan, J. R. (2001). Motor prediction. *Current Biology,**11*, R729–R732. 10.1016/S0960-9822(01)00432-8 11566114 10.1016/s0960-9822(01)00432-8

